# Chronic Recurrent Multifocal Osteomyelitis (CRMO) and Juvenile Spondyloarthritis (JSpA): To What Extent Are They Related?

**DOI:** 10.3390/jcm12020453

**Published:** 2023-01-05

**Authors:** Isabelle Koné-Paut, Inès Mannes, Perrine Dusser

**Affiliations:** 1 Kremlin Bicêtre Hospital, DMU Child and Adolescent Health, Pediatric Rheumatology, CEREMAIA, University of Paris-Saclay, AP-HP, 94270 Le Kremlin-Bicêtre, France; 2 AP-HP, Kremlin Bicêtre Hospital, DMU 14 SMART IMAGING, Pediatric Radiology, 94270 Le Kremlin-Bicêtre, France

**Keywords:** chronic recurrent multifocal osteomyelitis, chronic nonbacterial osteitis, juvenile spondyloarthropathy, pathophysiology, genetics

## Abstract

Chronic recurrent multifocal osteomyelitis (CRMO) is an autoinflammatory disease occurring mainly in the pediatric age group (before 16 years) and generally presents as a separate entity. Synovitis, acne, pustulosis, hyperostosis and osteitis (SAPHO) syndrome combines osteoarticular and cutaneous involvement, similar to CRMO, and falls into the spectrum of spondyloarthritis (SpA). The fact that a patient can progress from one disease to another raises the question of whether CRMO, like SAPHO, could fall within the spectrum of SpA, ranging from a predominantly osteoarticular form to an enthesitic form with more or less marked skin involvement. In this review, we set out to discuss this hypothesis by highlighting the differences and similarities between CRMO and juvenile SpA in clinical, radiological and pathophysiological aspects. A common hypothesis could potentially consider intestinal dysbiosis as the origin of these different inflammatory diseases. Interindividual factors such as gender, environment, genetics and/or epigenetic background could act as combined disease modifiers. This is why we suggest that pathophysiology, rather than clinical phenotype, be used to reclassify these diseases.

## 1. Introduction

Chronic recurrent multifocal osteomyelitis (CRMO) and juvenile spondyloarthritis (JSpA) are two pediatric inflammatory diseases that have clinical similarities and lead to strong interference due to their respective extra-bony and extra-articular manifestations. The evolution from one to another is possible, and their therapeutic approaches are quite similar. Their pathophysiology is multifactorial and includes a complex genetic background closely related to innate immunity and the effects of a number of epigenetic mechanisms, as well as intestinal dysbiosis and stressor exposure.

## 2. CRMO

**Figure 1:**
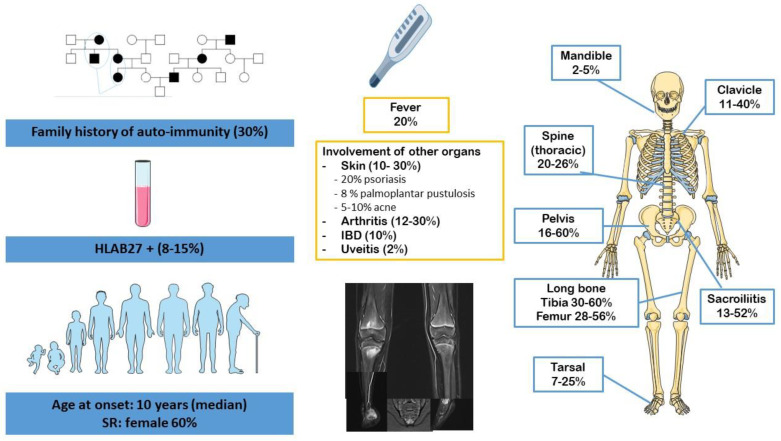
Clinical characteristics of chronic recurrent multifocal osteomyelitis (CRMO) [8].

CRMO is a primary inflammatory bone disease that affects the metaphysis of the long bones, the pelvis, the clavicle and the spine [1]. The incidence and prevalence of CRMO are not well-known (0.5–6/1,000,000 children) [2] and are probably underestimated. CRMO presents with musculoskeletal complaints such as pain, tenderness and/or swelling at the affected sites, which are mainly the limbs. Given that it occurs in otherwise healthy children, it may be confused with growing pains. CRMO mainly affects children (median age of onset being 10 years) with a sex ratio favoring girls (approximately 2:1). It is characterized by chronic and recurrent episodes of osteoarticular inflammation. CRMO may be associated with skin rashes (psoriasis, palmoplantar pustulosis, acne conglobata) or digestive disease [3] (Figure 1). Biologically, the inflammatory syndrome is inconsistent and seems proportional to the number of affected sites. Whole-body magnetic resonance imaging (MRI) is the preferred imaging tool to diagnose and monitor CRMO [4]. Whole-body MRI has the advantages of avoiding radiation, demonstrating lesions confined to the marrow, excluding differential diagnoses, looking for complications (such as medullar compression in case of vertebral lesions) and assessing evolution under treatment. It provides information on the distribution of lesions and can demonstrate clinically occult sites of disease, confirming the multifocal nature of the disease. Hyperostosis is an integral part of the bone involvement of CRMO, and it can be a radiological and histological marker for diagnosis. Unifocal CRMO may require a bone biopsy, which can retrieve nonspecific inflammatory aspects and hyperostosis, and excludes infectious or malignant processes. The clinical heterogeneity of CRMO is obvious and concerns the age of onset, which can, in some rare cases, be very young (before the age of 2 years), the number of bone sites affected, the level of systemic inflammation, extraosseous involvement and the course of and response to treatment. The boundary with the SAPHO (synovitis, acne, pustulosis, hyperostosis and osteitis) syndrome, defined in adults by Kahn et al. [5] and revised in 2003 [6], remains unclear, with some children with CRMO developing palmoplantar pustular psoriasis and, more rarely, pustular cystic acne.

The course of the disease for any given patient is unpredictable; it is also difficult to monitor due to the lack of standardized tools. Ultimately, the disease may have mechanical repercussions due to the possible occurrence of osteoarticular deformities and limitations. Pain can have a major impact on quality of life. As a whole, prognosis is good, with a median duration of disease progression of 4 years and recovery without sequelae in the majority of cases [7].

## 3. Juvenile Spondyloarthritis

Juvenile spondyloarthritis (JSpA) may be defined as a heterogeneous group of diseases, with varying degrees of peripheral and axial arthritis and enthesitis and a strong association with human leukocyte antigen-B27 (HLA-B27), affecting children under 16. JSpA is a generic term that not only includes children meeting the criteria for juvenile idiopathic arthritis (JIA), categories of seronegative or seropositive enthesitis related arthritis (ERA) and juvenile psoriatic arthritis (JPsA), but also juvenile ankylosing spondylitis (JAS), reactive arthritis and inflammatory bowel disease (IBD)-associated arthritis [9]. ERA and JPsA account for most cases of JSpA, with ERA being the most common (50%). All the different forms of adult SpA may occur in children, but they usually present as undifferentiated SpA and progress to differentiated forms over time. In children, this group of diseases is characterized by enthesitis and peripheral arthritis, usually asymmetric, more or less associated with tenosynovitis and most often affecting the lower limbs [10]. A significant proportion of patients will develop arthritis of the sacroiliac and spinal joints during the course of the disease, and some will develop extra-articular manifestations involving the gut, eyes, skin and mucous membranes.

**Figure 2:**
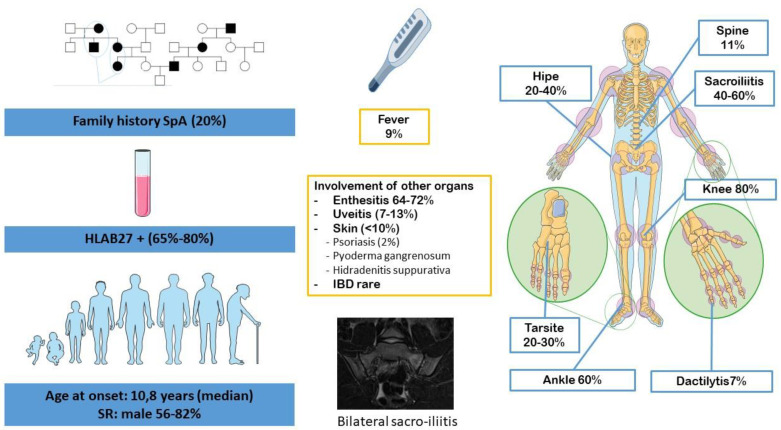
Clinical features of JSpA, specifically the ERA form of the JIA classification according to Edmonto et al. [14]. ERA: enthesitis-related arthritis; JIA: juvenile idiopathic arthritis; JSpA: juvenile spondyloarthritis.

ERA, or enthesitis/spondylitis-related JIA [11], refers to a group of human leucocyte antigen (HLA)-B27-associated inflammatory disorders affecting mainly male patients after 6 years of age (Figure 2). It represents 20% of all JIA. Unlike adult SpA, most patients start their disease with peripheral arthritis, predominantly oligoarticular (75%) and mainly affecting the lower limbs (such as the hips, knees, ankles and tarsal joints) [12]. Hip joint involvement predicts the development of sacroiliitis and is one of the major causes of morbidity and handicap in adulthood [13].

**Figure 3:**
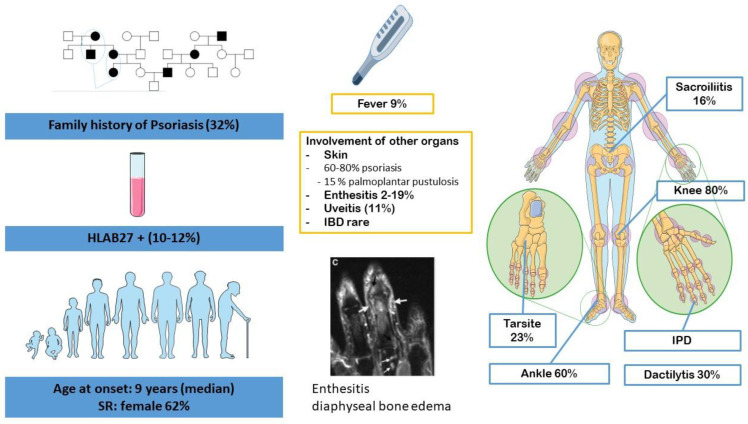
Clinical characteristics of JPsA. JPsA: juvenile psoriatic arthritis.

JPsA is rare (Figure 3). It accounts for 2–10% of all JIA and has two clinical phenotypes. The most often manifests itself in young girls of 2–3 years of age with either oligo- or polyarticular involvement with dactylitis and/or uveitis, with a high incidence of antinuclear antibody (ANA) positivity. The second is similar to the ERA JIA group and occurs most often in adolescent boys with enthesitis, sacroiliitis and psoriasis [15]. HLA-B27 is present in only 10.6% to 12.0% of cases, with a similar distribution in early-onset and late-onset JPsA, and does not correlate with axial involvement [16].

Over the course of the disease, JSpA can evolve to a polyarticular form [16]. Delay in diagnosis is frequent because the initial signs, i.e., lower back and heel pain, which is sometimes purely mechanical, are vague and misleading. Isolated enthesitis is another clinical trap that is difficult to distinguish from unexplained chronic pain syndrome [17]. Biological signs have no diagnostic value and systemic inflammation is variable and may even be absent. As in adults, girls are less affected. In addition, they are more likely to develop isolated enthesitis that is difficult to distinguish from unexplained chronic pain syndrome. In sum, with the exception of certain features at the onset of the disease, including an increased prevalence of peripheral disease and the rarity of axial symptoms, JSpA resembles adult forms of SpA in their association with HLA-B27, their clinical expression and their radiological features.

In the long term, most patients with JSpA remain active in adulthood; most often, patients are dependent on biotherapies. Older age of disease onset, HLA-B27 positivity, development of hip arthritis within the first 6 months and tarsitis are associated with a worse prognosis [18,19,20,21].

## 4. Links between CRMO and JSpA

### 4.1. Clinical Overlap

Is CRMO a distinct disease or does it belong to a spectrum of diseases including JSpA, as has been suggested for SAPHO syndrome by some authors [22]? Table 1 compares the clinical characteristics of the diseases.

#### 4.1.1. Bone Inflammation, Joint Involvement and Enthesitis

The clinical feature that differentiates pediatric CRMO from SpA is mainly the localization of inflammation. In CRMO, inflammation is mainly localized in the bones and affects the metaphysis of the long bones, especially in the lower extremities (78%) near the knees and ankles, as well as in the axial skeleton (48–68%) [9]. In SpA, the main target sites are the spine and the pelvic bones [24]. Sacroiliitis is most often bilateral and predominantly affects male HLAB27 patients (90%). In PsA, cervical involvement is the most common axial localization; sacroiliitis is less severe, unilateral with no sex ratio in favor of males and associated with HLAB27 in less than half the cases [25]. In contrast to SpA, axial involvement, especially spinal involvement, may be asymptomatic in CRMO, with incidental discovery through whole-body MRI [1].

SpA is also characterized by peripheral inflammatory arthritis associated with enthesitis, which is much less frequent in CRMO (12–30% [26] versus 87% [24]).

The mechanism underlying this inflammation is also to be considered when comparing these different diseases. Bone involvement is a common feature between SpA, especially PsA, and CRMO, but SpA seems to primarily affect the enthesis and to secondarily spread to the joints and bones [27]. Enthesitis is associated with marked osteitis or synovitis in the immediately adjacent tissues. MRI, with its potential to visualize both soft tissue and intraosseous abnormalities, has fostered our understanding of the entheseal organ concept by demonstrating the extension of enthesitis to adjacent bones and surrounding structures, including fibrocartilage, bursa, fat pad and deeper fascia [28]. Conversely, in CRMO, inflammation seems to originate primarily in the bone [29]. Vittecoq et al. suggested that CRMO could start at the enthesis and then evolve progressively toward inflammatory osteitis, explaining the link and future evolution toward SpA [22]. This hypothesis requires verification and should be viewed with caution.

#### 4.1.2. Gender, Genetic Background and Familial History

Unlike in JSpA, there is no male predominance and no strong association with HLA-B27 or family history of SpA in CRMO. In contrast, there is a strong family history of autoimmune disease (20–30%) in CRMO, as in PsA [30].

#### 4.1.3. Skin Involvement

Both JSpA, particularly JPsoA, and CRMO have skin involvement [12,26]. In both cases, psoriasis is present, although it is obviously more important in JPsoA.

### 4.2. Radiological Particularities of Each Entity

**Figure 4:**
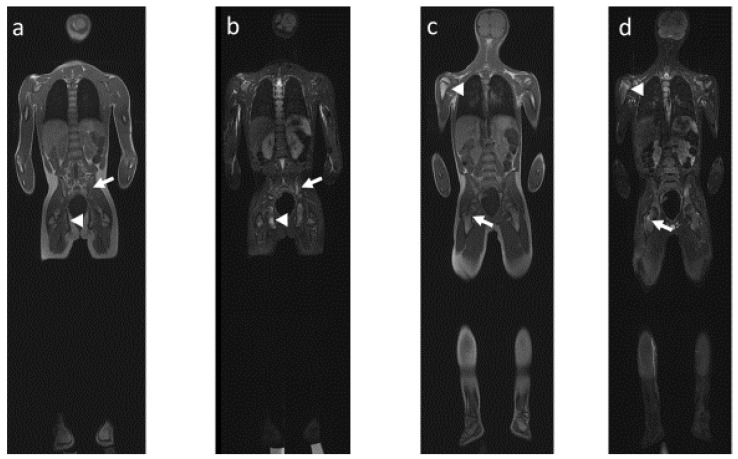
Whole body MRI, 12-year-old boy with confirmed CRMO. Coronal plane in (**a**) T1-weighted sequence and (**b**) STIR-weighted sequence showing bone marrow edema in the left iliac wing (arrow) and the right pubic ramus (arrowhead). Coronal plane (same exam) in (**c**) T1-weighted sequence and (**d**) STIR-weighted sequence showing bone marrow edema in the metaphysis of the right upper femur (arrow) and the metaphysis of the right upper humerus (arrowhead).

**Figure 5:**
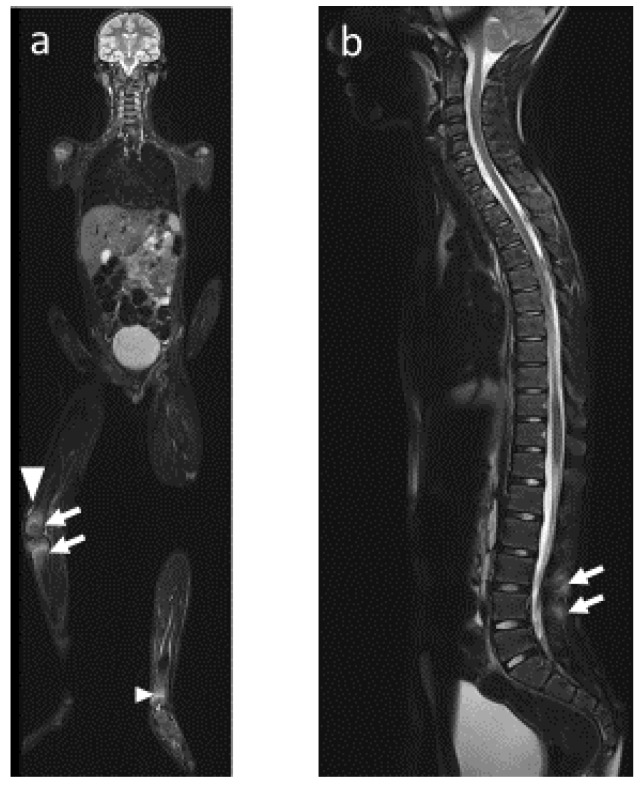
Whole body MRI, same patient 18 months later, CRMO with probable overlapping SpA. Asymmetrical position during MRI acquisition due to intense pain. Coronal plane in (**a**) STIR-weighted sequence showing bone marrow edema in the right lower femur and right upper tibia (arrow), associated with joint effusion (arrowhead) and bone marrow edema in the metaphysis of the left lower tibia (small arrowhead). Sagittal plane in (**b**) STIR-weighted sequence showing periosseous edema in the interspinous space (arrows).

Schematically, as explained previously, CRMO mainly consists of osseous lesions, whereas JSpA first includes joint anomalies, leading secondarily to osseous changes. In both diseases, MRI allows the visualization of most of the osteoarticular changes, including (1) bone marrow lesions, especially when using coronal T1-weighted and STIR sequences in addition to axial STIR images (Figure 4 and Figure 5), and (2) inflammatory lesions of the axial joints, which are best visualized on T1 sequences and include erosion, sclerosis, fat metaplasia, backfill and ankyloses [31].

CRMO, SAPHO and JSpA can show similarities in radiological findings (Figure 4 and Figure 5).

(1) Bone lesions

In JsPA, periosteal appositions and enlargement of the epiphyses are frequent. Joint-space narrowing and osseous erosions can also be observed. MRI may also show two types of lesions: first, inflammatory lesions such as bone marrow edema (BME) close to the enthesis and in the diaphysis [32,33]; second, structural lesions such as bone erosion or bone proliferation [34]. In CRMO, radiographs can show lytic, increased density or mixed density lesions, but lesions can appear as bone edema, periosteal reaction, hyperostosis, osteolytic with a sclerotic rim, mixed lytic and sclerotic lesions, purely sclerotic lesions, vertebral compression and soft tissue involvement [35].

(2) Vertebral and paravertebral involvement

Spinal manifestations of SpA can present with BME in the vertebral bodies [32,33], but also as paravertebral ossification and angular lesions. Ossifications usually consist of marginal and symmetric syndesmophytes/parasyndesmophytes in AS and nonmarginal and asymmetric syndesmophytes/parasyndesmophytes in PsA [36]. Corner lesions of the spine can also be seen in JSpA, manifesting as erosions, marrow oedema or osteitis at the end plates of the vertebral body [37]. These lesions probably indicate enthesitis and precede the development of fatty corner lesions, as well as the formation of syndesmophytes. Vertebral lesions in CRMO (around 30%) are typically multifocal and discontinuous, and they mostly affect the thoracic spine [38,39]. Paravertebral ossification has not been described in children with CRMO, only in adult SAPHO syndrome similar to that described in PsA. Finally, the spondylodiscitis of CRMO is equivalent to the aseptic spondylodiscitis occasionally seen in patients with AS [29].

(3) Sacroiliac involvement

One third of CRMO patients will develop an iliac lesion [40]. These mostly affect the pelvic bones adjacent to the sacroiliac joint, the triangular cartilage or the ischiopubic symphysis. In PsA syndrome, sacroiliac joint involvement is usually unilateral and can be erosive with extensive sclerosis of the adjacent iliac or sacral bone. In JSpA, it typically manifests itself as symmetrical sacroiliitis (40–60%).

(4) Mandibular involvement

Posterior mandibular involvement is specific to CRMO; it has never been described in JSpA. They are characterized by osteolytic lesions with associated variable amounts of periosteal new bone formation causing hyperostosis and a variable degree of sclerosis [29]. Only the temporomandibular joint has been described as affected in JSpA [29].

### 4.3. Pathophysiological Particularity: Differences and Similarities 

**Figure 6:**
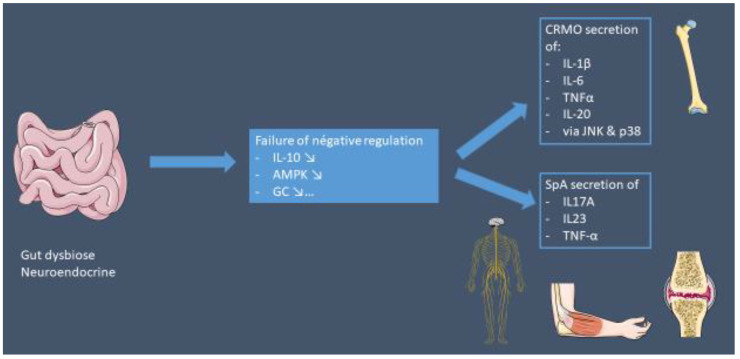
Similarities between CRMO and SpA. Intestinal dysbiosis may be the common factor in the expression of these two conditions. Depending on the genetic predisposition (HLAB27, NLPR3 methylation, ERAP1, etc.) and the environment, this intestinal inflammation could be the cause of chronic inflammation.

The pathophysiological differences and similarities have been summarized in Figure 6.

#### 4.3.1. IL17/23 Axis

##### Spondyloarthritis

Although the pathogenic mechanisms underlying JSpA (especially AS and PsA) are not fully elucidated, much evidence suggests that interleukin 17A (IL-17A) plays a pivotal role in these diseases [41]. In healthy individuals, IL-17A, as well as other members of the IL-17 family, functions in host defense against a range of bacterial and fungal pathogens at epithelial and mucosal barriers in the skin, colon and airways [41]. IL-17A plays a role in SpA manifestations related to the skin, joints and enthesis, as reflected by the suppression of disease activity seen with IL-17A inhibitors in psoriasis, PsA and AS [42,43,44,45,46,47,48,49]. However, in other settings where IL-17 family members have been found at sites of disease, such as gut inflammation and uveitis, IL-17A inhibition is not beneficial [50,51,52]. These discrepant responses illustrate the need for a clearer understanding of the role of the IL-17 family in the context of the tissue(s) affected.

##### CRMO

The pathophysiology of CRMO is unclear. However, a genetic predisposition seems likely, given the few descriptions of families with CRMO and/or the high incidence of cutaneous (psoriasis) and/or digestive (IBD) autoimmune diseases in patients and/or first-degree relatives (50%) [23,53]. CRMO results from an imbalance between pro- and anti-inflammatory cytokines [54,55,56,57]. Monocytes from patients with CRMO do not express IL-10, an anti-inflammatory cytokine, in response to stimulation of the Toll-like receptor (TLR) 4 by lipopolysaccharides (LPS) [55]. This alteration results, among other things, from a defect in the mitogen-activated protein kinase (MAPK) signaling pathway: ERK1 and 2 [54]. In contrast, the proinflammatory pathways, mediated by Jun kinase (JNK) and p38MAPK, function normally. Inflammatory cytokines (TNFα, IL-6, IL-1β and IL-20) are therefore produced and are not compensated for by anti-inflammatory cytokines, favoring a proinflammatory state. This inflammation has an effect on the bone, as it increases the interaction of RANK (receptor activator of nuclear factor-κB) membrane receptors with their soluble ligand RANKL on osteoclast precursor cells and induces osteoclast differentiation and activation [26].

#### 4.3.2. Intestinal Inflammation: The Common Link between CRMO and JSpA?

Subclinical gut inflammation has been reported to be present in both JspA and CRMO [58,59,60]. Disruption of the epithelial layer brings gut microbes into direct contact with host immune cells, activating an aberrant inflammatory response that reaches joints or bones.

In CRMO, Rausch et al. showed an association between the presence of the HACEK group of bacteria (Haemophilus parainfluenzae, Aggregatibacter actinomycetemcomitans, A. aphrophilus, A. paraphrophilus, *Cardiobacterium* spp., *Eikenella corrodens*, *Kingella* spp.) and disease activity [60]. In addition, Aggregatibacter is known to disrupt neutrophil membrane integrity via leukotoxin A (LtxA) and thus reduce and deregulate neutrophils, which may be the causative agents of inflammation in animal models of CRMO via (pro-)IL-1β [61,62]. Cardiobacterium is also thought to increase IL-1β levels [63] and to be associated with SAPHO syndrome, although this is controversial [64], possibly suggesting that these HACEK bacteria may play a role in bone inflammation in CRMO and SAPHO [60].

The involvement of the gut–articulation axis in the inflammation of JSpA is supported by the clinical success of anti-TNF and anti-IL-23 therapies in IBD and in some forms of SpA [65]. In adults with HLA-B27-positive early AS, dysbiosis and leaky gut lead to adaptive immune activation, which is associated with the characteristic MRI phenotype of osteitis [66]. Data from animal models and studies of relatives of AS patients strongly suggest that these changes do indeed precede the onset of the disease [67]. In contrast to CRMO, where hyperactivation of the NLRP3 inflammasome and, thus, hyperproduction of IL-1β is thought to be the cause of bone inflammation, in AS, this hyperactivity was found to maintain gut homeostasis and thus protect against gut inflammation [68]. It appears that remodeling of the gut microbiota and increased induction of regulatory T cells are the main mechanisms responsible for the observed resistance [69]. Therefore, defective NLRP3 inflammasome signaling in the gut is thought to contribute to IBD, causing intestinal leakage and the induction of detrimental immune responses against commensal invaders [70].

#### 4.3.3. Innate Immunity: A Common Thread?

The hypothesis is that both CRMO and JSpA originate from a deregulation of the innate immune system via a digestive and neuroendocrine axis [67], with secondary involvement of the adaptive system in the case of JSpA. The consequence would be a hypersecretion of IL-1 in CRMO and an activation of the IL-23/IL-17A pathway in JSpA. The role of innate immunity in the pathogenesis of SpA has recently been questioned, and some tend to classify SpA as a polygenic autoinflammatory disease in which the IL-23/IL-17 pathway plays a major role [71]. Several factors support this theory. Firstly, IL-23 could be secreted in susceptible individuals through HLA-B27 misfolding via the unfolded protein response or autophagy processors. IL-23, as well as the detection of B27 dimers by Killer Immunoglobulin Receptors (KIR) (NK cell receptors involved in MHC class I recognition and overexpressed in SpA patients compared to controls), induces IL-17 production via IL-23R positive cells. Secondly, whole genome expression profiling suggests a deregulation of the TLR pathway in peripheral blood and mesenchymal stem cells (MSCs) of AS patients [72,73]. It has been proposed that macrophages from AS patients express less IFN-γ, thus overexpressing genes usually underexpressed by this cytokine and vice versa [74]. Lower levels of this cytokine could promote the Th17 response and have a positive feedback mechanism on M2 macrophages [75], which, in turn, release higher amounts of IL-6, a key cytokine in the polarization of IL-17-producing cells.

#### 4.3.4. What about the HLAB27

The frequency of familial aggregation and the susceptibility due to HLA-B27 make JSpA a highly heritable disease, although many other factors influence its clinical expression and course. Indeed, HLA-B27 is strongly associated with the development of JSpA, although the prevalence of HLA-B27 in the disease in adulthood is higher [76]. However, the exact role of HLA-B27 in the pathogenesis is still unknown, as no more than 5% of HLA-B27+ individuals develop SpA, suggesting the involvement of other genetic and environmental influences. HLA-B27 has been shown to be both a risk factor and a severity factor for JSpA, particularly for diseases occurring in males and with axial involvement. Its role in CRMO is more debatable, and the largest patient cohort from the EUROFEVER registry reported 7% HLA B27 positivity [71], which approximates the general population. However, the presence of HLA B27 in CRMO could be an important modifying factor causing the development of SpA. HLA-B27-positive CRMO patients showed higher numbers of lesions clinically as well as radiologically, with a particular involvement of six or more bones [77]. In the 774 patients described by Reiser and al., 4.6% of HLA-B27-positive CRMO patients, compared to 2.4% of the whole cohort, had a codiagnosis of ERA JIA. HLA-B27-positive CRMO patients had a significantly higher involvement of the tarsal bones, including the calcaneus. Therefore, Reiser et al. assumed that HLA-B27 presence and CRMO affecting the tarsal bones may be a prognostic marker for the development or codiagnosis of JSpA [76]. In contrast, Vittecoq et al. described SpA evolution in a small French cohort without the presence of HLA-B27 [22].

### 4.4. Similar Therapeutic Approaches

There are also similarities in terms of treatment strategies. Indeed, both anti-TNF and anti-IL-17 have shown efficacy for these different diseases, which we could consider as an additional indirect argument in favor of a certain similarity [64,65,66,67,68]. It is possible, however, that TNF blockade interrupts inflammatory pathways in a nonspecific manner, whether or not the diseases have common pathophysiologic mechanisms.

## 5. Discussion

In sum, can we try to answer the question of whether CRMO, like SAPHO, could be part of the JSpA spectrum, as King et al. suggested in 1987 [78]? This question is not easy today. Nevertheless, this review has allowed us to identify a link between CRMO and the JSpA group and, in particular, with common seronegative SpA (AS, reactive and psoriatic arthritis, ERA and arthritis associated with inflammatory bowel disorders) because of the high frequency of axial involvement (spinal lesions and sacroiliitis), the rather similar imaging findings and the pathophysiological similarities, despite obvious differences. In addition, two other arguments support this hypothesis. The first is the possible evolution from CRMO to SpA. However, this is atypical SpA because of the absence of a family context, the predominance of females, the often unilateral sacroiliac involvement and the absence of a link with the HLA-B27 haplotype. The second argument is the identification in the literature of similarities between pediatric CRMO and adult SAPHO (an entity already included by most in SpA) due to its similarities in unifocal and multifocal involvement, axial lesions and extraosseous manifestations [79]. In SAPHO, the main target sites are the anterior chest wall (sternum and the sternoclavicular, manubriosternal, costosternal and costochondral junctions) followed by the spine and pelvic bones [23]. Multiple or symmetrical lesions of the long bones are found in SAPHO (30%), as in CRMO [24]. Sacroiliitis affects both CRMO and SAPHO patients (13–52%), most often with unilateral involvement [79]. Finally, both SAPHO and CRMO have skin involvement similar to that of PsA [30].

The response of SAPHO patients to IL-17A blockade may confirm the existence of variable pathophysiological mechanisms in adult SAPHO patients compared to children with CRMO; however, we know nothing about the blocking effect of IL-17 in CRMO. Given that some SAPHO patients may have started with isolated CRMO before the onset of skin lesions, it is questionable whether the involvement of T cells in SAPHO syndrome is related to a dysregulation of the adaptive immune system per se or a secondary activation of the adaptive immune system [80].

For all these reasons, we suggest that there may be a continuum between these different entities (JSpA, SAPHO syndrome and CRMO), ranging from a purely bony form (CRMO) to more enriched phenotypes with more or less marked skin, enthesitis and spinal involvement. Table 2 summarizes the different characteristics of these different diseases. We think that an approach directed toward physiopathology that takes into account the genetic context is the best method to link these various diseases. This different way of looking at this spectrum of diseases could better prevent the evolution of the diseases and optimize treatment strategies.

## 6. Conclusions

CRMO share many features with SpA; thus, we can hypothesize that these conditions are part of the same spectrum, with no distinct categories but rather a number and gradation of symptoms observed under different conditions. Thus, we anticipate that classifications based on the underlying disease process will progressively replace current classifications based on similarity of clinical features.

## Figures and Tables

**Table 1:** Clinical characteristics of CRMO [23] and SpA [12,24].

	CRMO	SpA
SR (boys/girls)	0/5	1/7
Age at onset (years)	10	9, 5
Fever	17–20%	9%
Axial involvement	60%	63%
Dorsal spine	24%	24%
Lumbar spine	rare	44%
Sacroiliac joints	13–52%	47%
Enthesis	18–33%	86%
Peripheral arthritis	12–30%	87%
HLAB27	10%	38–68%

**Table 2:** Comparative table of clinico-radiological characteristics of JSpA, CRMO and SAPHO syndrome.

	JSpA		SAPHO	CRMO
	ERA	JPsA		
Clinical Manifestations				
Axial	Inflammatory back pain	Inflammatory back pain Asymptomatic	Anterior chest wall pain Soft tissue swelling Pain in the spine or gluteal region	Musculoskeletal complaints such as pain, tenderness and/or swelling plus hyperostosis of the medial end of the clavicle
Peripherical	Arthritis of the large joints	Polyarticular involvement	Long bone pain (30%) Peripheral arthritis (12–60%)	Long bone pain (30%) Peripheral arthritis (12%) Clavicular lesion
Enthesitis	64–72%	18–33%	13–50%	18–33%
Skin involvement	Psoriasis 2%	Psoriasis 60–80% PPP 14.6%	PPP 90% Psoriasis 8–16% Acne 5–10%	Psoriasis 20% Acne 5%
Imaging	Symmetrical sacroiliitis (40–60%) Symmetrical and marginal syndesmophytes in adults	Cervical involvement Unilateral sacroiliitis (less severe) Non marginal syndesmophytes and paravertebral ossification in adults	Anterior chest wall (65–90%) Spinal lesion (33%) thoracic spine > lumbar spine > cervical spine Unilateral sacroiliitis (13–52%): sclerosis and hyperostosis on the iliac side of the joint Long bone involvement (5–10%) Mandibular lesions (1–10%) Paravertebral ossifications: non marginal and asymmetrical syndesmophytes	Metaphysis of tubular bones Vertebral lesion of the thoracic spine (30%) Inflammatory osseous changes in pelvic bones adjacent to the sacroiliac joint Mandibular lesions (5%)

PPP: palmoplantar pustulosis; JPsA: juvenile psoriatic arthritis; ERA: enthesitis-related arthritis; JSpA: juvenile spondyloarthritis; CRMO: Chronic recurrent multifocal osteomyelitis; SAPHO: synovitis, acne, pustulosis, hyperostosis and osteitis syndrome.

## Data Availability

Not applicable.
